# Focus on organic electronics

**DOI:** 10.1088/1468-6996/15/4/040301

**Published:** 2014-08-08

**Authors:** Alexander Wei, Yasunari Zempo

**Affiliations:** 1 Purdue University, USA; 2 Hosei University, Japan

Electronically active materials, once largely the domain of inorganic metals and semiconductors, have become increasingly organic in character. Contemporary electronic materials such as light-emitting device (LED) displays, photovoltaic cells, and thin-film transistors (TFTs) all feature organic molecules with *π*-conjugated backbones, whose processability and mechanical flexibility present opportunities not available to traditional inorganic materials. For example, the device structure of organic LEDs is robust yet simple in design, comprised of a light-emitting layer of conjugated polymers between cathode and anode, while producing emissions at low voltages and energy consumption. Simply by manipulating the substituents along the molecular backbone, *π*-conjugated systems can also be tuned to emit at specific wavelengths, and can even be designed for white light emission. The commercial development of these applications has been quite rapid: at the time of this writing, organic LED displays are now available with areal dimensions of 1–2 m^2^ and resolutions up to 400 ppi.

The successful rise of organic electronics has been driven by several seminal contributions, two of which have been recognized with awards of the Nobel Prize in Chemistry: one in 2000 to Heeger, MacDiarmid, and Shirakawa for the discovery of conducting polymers, the other in 2010 to Heck, Negishi, and Suzuki for the development of transition-metal mediated cross-coupling, the synthetic basis for many of today’s *π*-conjugated polymers. Nevertheless, there are many opportunities for significant advances in scope as well as in performance, and for the scalable integration of organic electronic materials with inorganic substrates. Solid-state lighting technology represents one such area; electroluminescent organic materials are now widely available, and recent developments have produced organic materials with novel actuation properties such as thermally activated (delayed) fluorescence and polarized light emission. Many of these discoveries began with the design of small molecules, then developed into polymers with pliable backbones or extended *π*-conjugation, to facilitate their processing into organic LEDs and photovoltaic materials. The understanding and design of materials properties is aided by intensive computational studies, powered by ongoing developments in information technology. From these perspectives, organic structures will continue to play important roles in the design of next-generation electronic materials.

Efficient organic electronic devices depend on more than molecular structure. For example, mechanisms of photoactivated transport need to be elucidated in order to improve the efficiency of organic TFTs and photodiodes. Device optimization also requires us to understand the fundamental physics of charge injection, transport, and recombination between layers, and also between layers and substrate. Effective engineering of these interfaces requires intensive investigations in both materials design and layer integration—topics of significant interest in this issue of *Science and Technology of Advanced Materials*.

We would like to express our thanks to all authors who contributed to this focus issue on organic electronics, and hope that its contents will serve both as a digest and a catalyst for exciting research activities in this direction.

